# Chronic health conditions and school performance in first graders: A prospective cohort study

**DOI:** 10.1371/journal.pone.0194846

**Published:** 2018-03-27

**Authors:** Isabell Hoffmann, Christiane Diefenbach, Christine Gräf, Jochem König, Martina F. Schmidt, Kathleen Schnick-Vollmer, Maria Blettner, Michael S. Urschitz, for the ikidS Study Group

**Affiliations:** 1 Division of Paediatric Epidemiology, Institute of Medical Biostatistics, Epidemiology, and Informatics (IMBEI), University Medical Center of the Johannes Gutenberg University, Mainz, Germany; 2 Division of Epidemiological Methodology and Radiation Research, Institute of Medical Biostatistics, Epidemiology, and Informatics (IMBEI), University Medical Center of the Johannes Gutenberg University, Mainz, Germany; University Children's Hospital Tuebingen, GERMANY

## Abstract

**Objective:**

Children with chronic health conditions may perform poorer at school. Associations may be confounded by numerous social factors. We aimed to estimate the effects of a chronic health condition on overall school performance in first graders with an emphasis on rigorous adjustment for potential confounders.

**Methods:**

A population-based cohort study was performed in the area of Mainz-Bingen (Germany). In 2015 all preschoolers were approached and the presence of a chronic health condition was assessed by parental questionnaires and preschool health examination data. The identification of a chronic health condition was based on special health care needs and presence of a doctor’s diagnosis out of 24 school-relevant diseases. At the end of the first school year, overall school performance was assessed by teachers and rated on a 5-item scale ranging from -10 to +10.

**Results:**

Of 3683 children approached, 2003 were enrolled. Overall school performance was available for 1462 children (51% boys). Of these, 52% suffered from a chronic health condition. Compared to children without a chronic health condition, children with special health care needs (15%) performed worse at school (adjusted mean difference: -0.95, 95% CI: [-1.55; -0.35], *P* = 0.002). Children with a doctor’s diagnosis but without special health care needs (37%) did not perform worse at school. The effect was further analysed considering the extent of special health care needed.

**Conclusions:**

Chronic health conditions affect overall school performance early in primary school. To identify academically at-risk children, a chronic health condition identification based on special health care needs may be used.

## Introduction

There are many reasons that children underperform at school, including a poor socio-cultural (home) environment, specific learning disabilities, and in particular chronic health conditions (CHC). Since 2005, several systematic reviews have investigated and evaluated the growing body of evidence in this field [1–4]. In 2011, Suhrcke and de Paz Nieves were the first to propose a theoretical framework for the relationship between CHC and educational outcomes [5]. They differentiated between short- (i.e. academic performance) and long-term (i.e. educational attainment) outcomes, proposed potential mediating factors and confounders, and presented cross-sectional and longitudinal evidence for the relationship. Dadaczynski was the first to include only longitudinal studies to reach a higher level of causal inference in his systematic review [6]. He pointed out that adequately designed and analysed longitudinal studies are lacking. In summary, these reviews provided supportive evidence that CHC such as asthma, obesity, sleep disorders, and mental health disorders (i.e. anxiety, depression, behavioural disturbances, and attention-deficit hyperactivity disorder) may have a negative impact on educational outcomes. The authors underlined the importance of appropriate adjustment for confounding factors such as socio-economic status and pointed out that the level and quality of health care prior to school entry as well as the extent and effectiveness of special educational support have not been sufficiently investigated in most studies [5, 6]. However, this is important to understand regional differences in the effects of CHC on educational outcomes and to identify ameliorating effects of health care interventions and special educational support on educational outcomes.

There is a large debate on the appropriate framework of CHC in childhood [7]. The identification of a CHC can be based on either a specific doctor’s diagnosis of a chronic disease (i.e. diagnosis-based framework) or the need for special health care services (i.e. consequence-based framework). The diagnosis-based framework is grounded in the ICD classification, the availability of valid methods for the diagnosis, a non-curability of the disease, and the actual or expected duration of the disease [8]. The consequence-based framework is usually operationalised in short parental screening questionnaires such as the Children with Special Health Care Needs (CSHCN) Screener [9]. This instrument is increasingly used in national and international studies on epidemiological aspects of CHC in childhood [10–12]. However, studies combining both frameworks are lacking. In particular, it is unclear which framework should be used to identify academically at-risk children prior to school entry.

We, hence, initiated the ikidS project (ikidS: **i**ch **k**omme **i**n **d**ie **S**chule [German]; I am starting school) in 2013 as the first large cohort study in Germany investigating long-term outcomes of CHC in primary schoolchildren. The project combined both frameworks of CHC, investigated outcomes in the domains mental health, quality of life, social participation, and school performance, and assessed the use of health care services as well as special educational support. In the present report, we aimed to estimate the effects of CHC on 1^st^ grade overall school performance (OSP) in combining both frameworks of CHC and with rigorous adjustments for potential confounders.

## Methods

### Study region, setting, and design

A prospective, population-based, closed cohort study was performed between September 2014 and September 2016 within the city of Mainz and the district of Mainz-Bingen (Federal State of Rhineland-Palatinate; Germany), which covers an area of 704 square kilometres with a population of 418,528 inhabitants. In 2015, there were 79 public and private primary and special needs schools located in this area, and 3,683 children were officially registered for their first year of school. The research protocol was approved by the ethics committee of the regional medical association of Rhineland-Palatinate, the regional supervisory school authority, and the state representative for data protection in Rhineland-Palatinate.

### Study population, enrolment, and study sample

The study population consisted of all children who had their preschool health examination (PHE) within the study region between September 1^st^, 2014, and August 31^st^, 2015. The PHE is a standardised, compulsory, state-wide health examination performed by public youth health physicians employed by the regional Department of Public Health of the Mainz-Bingen District. Participants were enrolled on the day of the PHE by a public youth health physician and parental informed written consent was obtained. For the present work, children, who i) ultimately did not enter school in September 2015, ii) were assigned to a special needs school or iii) had no results for 1^st^ grade OSP were later excluded from the analysis.

### Procedures and data collection

Data were collected at four time points: at the PHE during the last preschool year (T0), six weeks before school entry (T1), three months after school entry (T2), and at the end of 1^st^ grade (T3). The PHE comprised a statutory parental questionnaire (including information on parental education and migrant background), a thorough medical history and physical examination, and the administration of screening tests as well as tests on pre-academic skills. At time points T1 to T3, general and mental health, presence of CHC, need for and use of special health care, family structure and burden, leisure time activities, nutritional habits, environmental conditions, and socio-economic status were assessed by using study-specific parental questionnaires, respectively.

### Identification of children with CHC

The identification of children with CHC based on both the consequence-based and the diagnosis-based framework. For the consequence-based approach, children with actual needs for special health care services were identified by a German version of the CSHCN Screener [9]. The CSHCN Screener is a 14-item parent-reported instrument covering five aspects (or indicators) of medical care: (1) need for prescription medication, (2) need for social or educational support, (3) functional limitations, (4) need for physical, occupational, or speech therapy, and (5) mental health problems. Each aspect has one key question with one or two additional questions, which assess whether the condition is due to a medical or other health condition and whether the duration or expected duration of the condition is 12 months or longer. The Screener indicates a CHC if the key question and its accompanying question(s) are answered positively for at least one of the five aspects. Validity and performance of the Screener have been investigated in children with disabilities [9] and diagnoses of chronic conditions made by a physician [11] as well as with respect to other parental screening instruments [13]. The Screener has been used in nation-wide health surveys in the US [14] and Germany [10]. In the present study, the CSHCN Screener was administered at time points T1 to T3.

Regarding the diagnosis-based approach, PHE data and study-specific parental questionnaires were evaluated at time points T0 to T3 for the presence (“ever”, “during the last 12 months” or “currently”) of one of the following 24 doctors’ diagnoses or chronic conditions: anaemia, asthma, atopic dermatitis, attention-deficit hyperactivity disorder, autism, conduct disorder, emotional problems, depression, diabetes mellitus, dwarfism, epilepsy, heart defect, hay fever, hearing impairment, hypothyreosis, low birthweight, overweight/obesity, premature birth, sleepiness, snoring, speech and language disorder, tumour/cancer, being underweight, and visual impairment. For these diagnoses and conditions, associations with educational outcomes have been demonstrated in previous studies [1, 5, 15–30]. A detailed description of how these diagnoses and conditions were identified is given in S1 Table.

### Educational outcomes

OSP was defined in accordance with the German National Educational Panel Study [31]. Teachers were asked to rate the child’s abilities in reading, writing, numeracy, science, and in social competencies in comparison to their peers of same age, each on a 5-point rating scale ranging from “much lower than average” and “lower than average” to “in average”, “higher than average” and “much higher than average”. Arbitrary numerical scores were assigned to each response category (i.e. -2, -1, 0, +1 and +2, respectively). Item scores were summed to obtain a combined OSP score ranging from -10 to +10, with 0 indicating average performance.

### Statistical methods

After enrolment of the study participants, representativeness of the sample was assessed by comparing important health and socio-demographic characteristics between the study sample and the source population. Anonymous PHE data provided by the Department of Public Health were analysed using appropriate statistical parameters (e.g. numbers and frequencies for categorical variables, and mean and standard deviation (SD) for normally distributed variables).

For the primary analysis, the exposure variable was CHC as defined in Table 1. In terms of simpler legibility, we call the reference group “no CHC”, even though there may still be children with special health care needs and/or doctors’ diagnoses. The primary outcome was OSP score. Potential confounders were identified following the causal model of Suhrcke and Paz Nieves [5] and an extensive literature search. Confounder variables were only included in the regression analysis if they were empirically associated in our data with both, exposure and outcome variables. The following confounders were ultimately included: socio-economic status, gender, migrant background, single parent family, multiple births (e.g. twins), breast feeding, household smoking, life style and leisure time activities (e.g. active and interactive behaviour, outdoor activities, early musical education, television in the child’s bedroom), and location of the school (see S2 Table). The following factors did not show an association with OSP and where not included in the model: single child, nutritional habits, CHC of a family member, and age at school entry.

**Table 1:** Definition of chronic health conditions (CHC) and frequencies within the study sample (N = 1462).

Chronic Health Condition	Definition	Frequency
Special Health Care Needs	(1) Children with special health care needs identified by the CSHCN^1^ Screener	171 (14.9%)
Doctor’s Diagnosis (only)	(2) Children with indications of one of the listed doctors’ diagnoses^2^ or conditions but without special health care needs as defined by the CSHCN^1^ Screener	423 (36.9%)
No Chronic Health Condition	Children not meeting criterion (1) or (2)	551 (48.1%)
Missing	Children with missing data concerning the CSHCN^1^ Screener	317

^1^ CSHCN–Children with Special Health Care Needs

^2^ List of doctors’ diagnoses and conditions: anaemia, asthma, atopic dermatitis, attention-deficit hyperactivity disorder, autism, conduct disorder, emotional problems, depression, diabetes mellitus, dwarfism, epilepsy, heart defect, hay fever, hearing impairment, hypothyreosis, low birthweight, overweight/obesity, premature birth, sleepiness, snoring, speech and language disorder, tumour/cancer, being underweight, and visual impairment.

The amount of missing values ranged from a very low frequency in variables assessed by PHE (e.g. gender: 0%, doctor’s diagnosis: 0%, smoking in household: 3%) up to 40% in variables assessed by study-specific parental questionnaires (e.g. early musical education: 20%, special health care needs: 22%, leisure time activities: 27%, socio-economic status: 40%). Missing values of the CSHCN Screener items and all confounders were imputed 10 times using chained equations with 100 iterations [32]. For each imputed variable, an individual set of variables was used as predictor variables. Missing values for OSP score were imputed using chained equations, if only one or two items of the OSP score were missing. In this case, the available OSP items were used as predictor variables. The effect of CHC on OSP was modelled using linear mixed-effects regression models with a random effect for school class and adjusted for the covariates mentioned above (M1). The results of the model are shown as effect estimates of CHC with 95% confidence intervals and *P* values. Results were also presented as least-means squares (marginal means) with confidence intervals for CHC stratified by gender.

As a secondary analysis, the relationship between special health care needs and OSP was further investigated by estimating the effects of each of the five individual indicators of the CSHCN Screener (M2), and the total sum of these positively answered indicators (M3) on OSP. For this analysis, a linear mixed-effects model with the same covariates and random effect for school class was used as in the primary analysis. The analysis was performed using the software packages *R* (version 3.4.1), *nlme* (version 3.1–131), and *mice* (version 2.30) [33] and the S1 Public Use File.

## Results

### Study participants and sample

**Fig 1:**
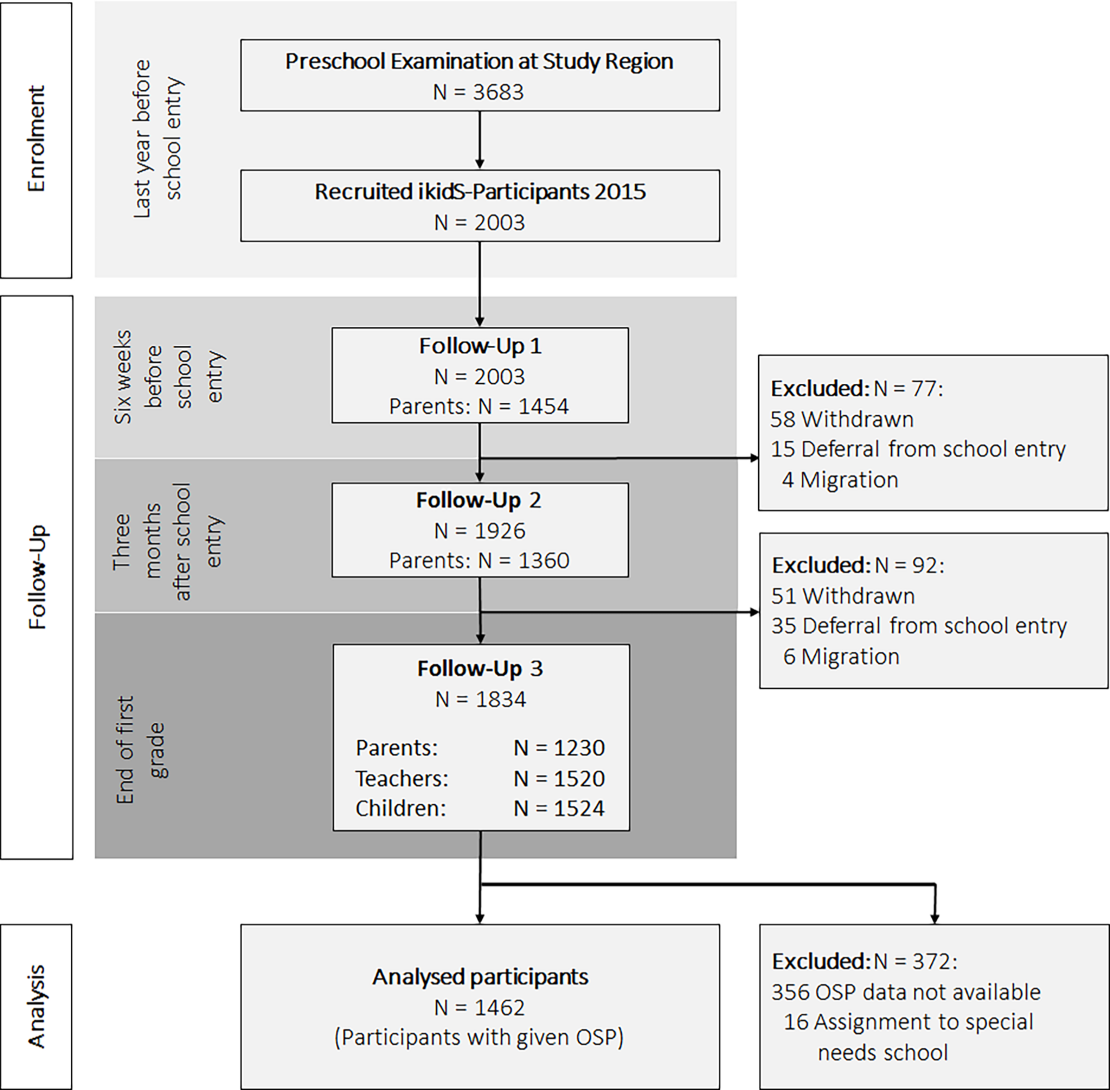
Flow-chart for the participants of ikidS, showing response rates of parental questionnaires, teachers’ and children’s assessments.

Of 3683 eligible children, 2003 (i.e. study participants, 54% of the study population) were enrolled. Responses of parental questionnaires ranged from 1454 at T1 to 1230 at T3, and 1524 children were assessed by teachers (Fig 1). Finally, diagnoses and OSP data were available for 1462 children (i.e. study sample, 40% of the study population; Fig 1). Except for migrant background (20.7% vs. 22.3%), the study sample was fully representative of the underlying study population (Table 2). However, outbalancing this deviation by using population-based sampling weights in the analysis (i.e. weighted regression) did not change results. Hence, data are not shown.

**Table 2:** Characteristics of children within the study region (population), children enrolled into the study (participants), and children with available school performance data (sample). Unless otherwise stated, characteristics are summarised by total numbers (N) and frequencies (%).

Characteristics	Study Population	Study Participants	Study Sample
	N = 3683	N = 2003	N = 1462
Child			
Male	1909 (51.9%)	1042 (52.0%)	751 (51.4%)
Migrant background	822 (22.3%)	452 (22.6%)	303 (20.7%)
Age at PHE^1^, mean (SD^2^)	5.86 (0.42)	5.89 (0.37)	5.89 (0.36)
Family			
Single parent family	344 (9.4%)	193 (9.7%)	134 (9.2%)
Multiples	105 (2.9%)	63 (3.2%)	50 (3.4%)
Abitur (A-level exams) Mother	1825 (60.4%)	1117 (60.7%)	827 (61.5%)
Abitur (A-level exams) Father	1768 (61.0%)	1042 (59.1%)	765 (59.3%)
Breastfeeding			
Not at all	573 (17.5%)	326 (17.0%)	231 (16.5%)
Up to 6 months	1312 (40.2%)	776 (40.4%)	574 (41.0%)
More than 6 months	1382 (42.3%)	817 (42.6%)	596 (42.5%)
Smoking in household			
Never	3033 (91.9%)	1764 (91.0%)	1293 (91.2%)
Seldom	191 (5.8%)	122 (6.3%)	94 (6.6%)
Often	77 (2.3%)	52 (2.7%)	31 (2.2%)
Health			
Speech and language disorder	567 (15.4%)	296 (14.8%)	200 (13.7%)
Visual impairment	295 (8.0%)	177 (8.9%)	128 (8.8%)
Atopic dermatitis	164 (4.5%)	99 (5.0%)	69 (4.7%)
Asthma	82 (2.2%)	46 (2.3%)	34 (2.3%)
School location			
Rural	1910 (51.9%)	1044 (52.1%)	783 (53.6%)
Urban	1773 (48.1%)	959 (47.9%)	679 (46.4%)

^1^ PHE–Preschool Health Examination

^2^ SD–Standard deviation

Information on doctors’ diagnoses was available for all study participants. The frequencies of the 24 diagnoses/chronic conditions under study are given in S1 Table. Of these, premature birth (N = 133), sleepiness (N = 119), atopic dermatitis (N = 113), asthma (N = 103), and being underweight (N = 84) were the most frequent CHC (S1 Table).

CSHCN Screener information was available for 1145 children (31% of the study population). Of these, 52% fulfilled at least one of the two criteria for CHC, whereby 15% were children with special health care needs, 45% had one out of the 24 diagnoses/conditions under study, and 37% had a diagnosis/condition but no special health care needs (Table 1). Need or use of prescription medication for an ongoing condition was the Screener criterion most frequently met by the children (N = 81; 6.9%), followed by above-routine use of health or related services (N = 79; 6.8), and treatment or counselling for an emotional, developmental, or behavioural problem (N = 72; 6.2%; S4 Table).

### Chronic health condition and overall school performance

Children with any kind of CHC had lower OSP scores (mean = 1.3; SD = 3.8) compared to children without CHC (mean = 2.1; SD = 3.4). Children with missing values for special health care needs (N = 317; 22% of the study sample) had an even lower OSP score (mean = -1.3, SD = 3.7), which demonstrated the need for imputing missing values for special health care needs. More information about the characteristics of the three CHC groups are given in S3 Table.

**Fig 2:**
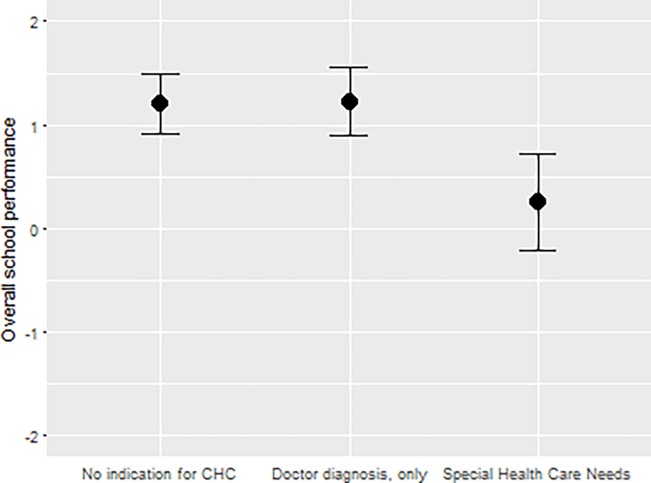
Least squared means with confidence intervals of OSP by CHC.

After imputing missing values and adjusting for potential confounders, children with special health care needs performed worse compared to children with no CHC (M1: adjusted OSP mean difference: -0.95, 95% CI [-1.55, -0.35], *P* = 0.002; Table 3). Children with one out of the 24 diagnoses under study but no special health care needs did not perform worse (M1 adjusted mean difference: -0.02, 95% CI [-0.38, 0.42], *P* = 0.98; Table 3). Fig 2 demonstrates the lower marginal means in OSP for children with special health care needs in comparison to the other CHC groups.

**Table 3:** Analysis of chronic health condition on overall school performance. The effect of chronic health conditions (M1), the effect of each indicator of the CSHCN Screener (M2) and the numbers of qualifying indicators of the CSHCN Screener (M3) on overall school performance as assessed by multivariable linear regression analysis (N = 1462).

		Overall School Performance^2^		
Model (M)	CHC^3^	Effect estimate^1^ (95% CI)		P value
**M1**: CHC effect model	Special Health Care Needs	-0.95	(-1.55, -0.35)	.002
	Doctor’s Diagnosis, only	0.02	(-0.38, 0.42)	.98
	No Chronic Health Condition	0 (Reference)		
**M2**: Indicators of the CSHCN Screener^4^	Indicator 1 –Use of prescription medications	0.33	(-0.50, 1.15)	.43
	Indicator 2 –Above-average use of medical, mental health, or educational services	-0.91	(-2.04, 0.22)	.11
	Indicator 3 –Functional limitations	0.02	(-1.34, 1.39)	.97
	Indicator 4 –Use of physical, occupational, or speech therapy	-0.88	(-2.00, 0.24)	.12
	Indicator 5 –Use of emotional, behavioural, or developmental treatment or counselling services	-0.58	(-1.63, 0.47)	.28
**M3**: Numbers of qualifying indicators of the CSHCN Screener^4^	0	0 (Reference)		
	1	-0.47	(-1.19, 0.25)	.20
	>1	-1.51	(-2.36, -0.65)	< .001

^1^ Effect estimates are pooled after imputing missing values by chained equations multiple times. Effect estimates are adjusted β-coefficients and represent the change in OSP associated with the independent variable, adjusted for the following confounders: socioeconomic status, gender, migrant background, socioeconomic status, one-parent family, multiples, breast feeding, smoking in house hold, active and interactive behaviour, outdoor activities, early musical education, television in child room, school location, and school class. Results of covariates included in the model are not shown.

^2^ Overall School Performance ranges from -10 (much lower than average) to 10 (much higher than average).

^3^ CHC–Chronic health condition

^4^ CSHCN–Children with Special Health Care Needs

### Indicators of the CSHCN Screener and OSP

The needs for special health care services was defined by 5 different indicators of the CSHCN Screener (S4 Table). Use of prescription medications (6.9% of those with available CSHCN Screener information) and above-average need for medical, mental health, or educational services (6.8% of those with available CSHCN Screener information) were the most common needs in the study sample.

In a secondary analysis, none of the single indicators of the CSHCN Screener showed a clear association with OSP (M2 in Table 3. However, if more than one indicator was fulfilled (7.2% of those with available CSHCN Screener information), OSP was markedly lower compared to children without CHC (M3: adjusted mean difference: -1.51, 95% CI [-2.36,-0.65], *P* < 0.001; Table 3).

## Discussion

In the present study, the presence of CHC at school entry was independently associated with OSP at the end of first grade. The association was clearly seen for children with increased needs of special health care and remained after adjusting for a series of potential confounders. Moreover, the observed effect was stronger in children, who had more than one indicator of special health care needs. To our knowledge, this is the first European study on this topic in primary schoolchildren, thereby compensating for the geographical bias in the research, which has been mentioned previously [5]. The data may now be used to further explore how investments in health care services could improve educational outcomes.

Our results are in line with previous studies in children and adolescents utilizing parental reports of special health care needs [34, 35], chronic illness [36], and doctors’ diagnoses of CHC [37, 38] as well as electronic health records of general practitioners as data sources of child health [39]. In contrast to these studies, we used two different and independent frameworks of CHC (i.e. diagnosis- and consequence-based) at the same time. In a previous study, the diagnosis-based framework yielded 285 different ICD diagnoses or clusters of diagnoses that fulfilled requirements for CHC [8]. However, in a public health setting (such as the PHE) it is not feasible to access such a large number of diagnoses. Instead, we restricted our assessment to diseases, which were frequent and relevant for school enrolment, management at school, and/or school performance. As a result, the presence of one out of 24 doctors’ diagnoses without increased special health care needs was not associated with OSP. This may question the benefit of generally assessing doctors’ diagnoses in the PHE. A low concurrence between parental reports and medical records in less educated parents further weakens the diagnosis-based framework for studies aiming at educational outcomes [40].

We found information of the CSHCN Screener independently associated with OSP. Children with two or more qualifying indicators of the instrument performed especially worse at school. This is in line with previous studies, where CSHCN Screener items and/or profiles were associated with educational outcomes such as school attendance, grade point average, special education needs, grade repetition, and lack of school engagement [34, 35]. Our results extend these observations and showed clear benefits of adding the CSHCN Screener to PHE. According to our results, this short instrument may be sufficiently accurate to identify an academically at-risk group of children prior to school entry. This may further replace parent reports of a doctor’s diagnosis in future developments of PHE.

Many studies have evaluated the association between one type or one group of CHC and educational outcomes [3, 5, 6, 41]. However, the cause-effect relationship between CHC and educational outcomes remains still unclear due to insufficient samples, inappropriate adjustments, and other methodological shortcomings in some studies [5]. There are likely many predictor variables, mediators and modifiers for educational outcomes in children and residual confounding remains a great threat to observational studies such as the present. Thus, we applied and extended an analytical framework for the relationship between health and education in high-income countries and for the selection of potential confounders [5]. They were evaluated for their association with CHC and OSP, and were finally included in the statistical models. Apart from socio-economic status and having a migrant background, we identified important family- and leisure time-related confounders. However, it is still possible that there is a third factor that explains the association.

In our study, the frequency of CHC ranged from 15% to 45% depending on the definition used (i.e. consequence- or diagnosis-based). There is a large range of definitions and operationalisations for CHC in childhood [7]. This leads to a wide variability in prevalence estimates, ranging from 0.2% to 44% [7]. Concerning the consequence-based definition, our results (15%) were in accordance with representative survey data from the German KIGGS study and the 2003 US National Survey of Children’s Health, which found prevalence estimates ranging from 13.7% to 17.6% [10, 12]. Concerning the diagnosis-based definition, our results (45%) were slightly higher compared to the KIGGS study (38.7%), which used a list of 17 common doctors’ diagnoses for the definition of CHC [42], and the Canadian Longitudinal Survey of Children and Youth (30.3%; [43]). The higher frequency in the present study could be explained by some items in the statutory parental PHE questionnaire that evaluated the presence of doctors’ diagnoses with a life-time perspective (i.e., items asked for “ever” having had a specific diagnosis). This has likely enlarged the group of children with any CHC but enabled us to form a largely “disease-free” reference group for unbiased estimations of CHC effects.

Several limitations should be mentioned. Early OSP was assessed by teacher reports at the end of first grade. As grades are not assigned to first and second grade pupils in Rhineland-Palatinate and objective and standardised school performance tests are not routinely performed in this age group, we used an established instrument from the German National Educational Panel Study to evaluate a broad range of school-related competencies. However, teacher ratings may be subjective to their occupational experience, their attitude towards a child, and the mean level of competencies within the whole class. The resulting clustering in the outcome variable has been accounted for in the analysis by adding a random intercept for teacher/class in the models.

The diagnosis-based approach was based on a list of doctors’ diagnoses, which were in the focus of the statutory PHE because these CHC were related to poor OSP in previous studies. However, the selection was more or less arbitrary and disease severity was not obtained. Hence, we might have neglected potentially important diseases that might have been relevant for OSP. In fact, CHC consist of a very diverse set of diagnoses. In the present study, we did not divide the conditions up in the analysis, because this would go far beyond the scope of the present publication. However, a breakdown of the CHC groups into different groups of diagnoses may well have shown vastly different results. Further analyses comparing mental CHC with physical CHC are currently underway and will enlighten the relative relevance of CHC groups in terms of educational outcomes. Until further results are available, we recommend focussing on those children who already have special health care needs according to the CSHCN Screener, independent of their specific diagnosis.

The consequence-based approach was based on the CSHCN Screener, a short, feasible, and efficient screening instrument developed to identify children with chronic conditions for a variety of purposes, including public health monitoring, health care quality assessment, and programme planning and evaluation [9]. The performance of the Screener varies depending on the setting and chronic condition. In a sample of children enrolled in the US Medicaid managed care health plans, 53% of children were screened as positive whereas in a sample of children receiving supplemental income for disability, 94% of children were correctly identified with special health care needs [9]. In two validation studies, sensitivity ranged from 69% for skin diseases to 91% for epilepsy [11], and the positive and negative predictive values were determined to be 93% and 89%, respectively [13]. However, children with CHC may not be identified as such as they do not meet the criteria of this Screener in every case [11]. Although the instrument is well established and frequently used in large surveys [10, 14], the precise accuracy of the instrument remains unknown, and due to its nature it is–at least to some extend—subject to misclassification.

There is vast variability within the category of CSHCN. Most children qualified for CSHCN due to a medication prescription (CSHCN indicator 1) or above-average use of medical care, mental health, or educational services (CSHCN indicator 2). The use of educational services is a marker of poor school performance and it would be not surprising that these children do not perform as well in school. However, only six out of 171 children were positively screened due to CSHCN indicator 2 at the end of the first grade. Excluding these children from the analysis did not change the effect estimates decisively. Thus, all children meeting the criteria of the CSHCN indicator 2 had in fact only above-average use of medical care or mental health services.

Missing values up to 40% of cases was an issue in this study, which can be a source of bias for effect estimates. In order to cope with that, missing values were imputed prior to regression analysis, which is an adequate method for dealing with values missing at random in longitudinal studies [44]. Although children with missing CSHCN Screener information had lower mean OSP values compared to children with available information, a complete case analysis resulted in the same effect estimates for CHC. The same source of bias was identified for socio-economic status. Hence, only effect estimates after imputation are presented in this work. This enabled us to present largely unbiased estimates of the association between CHC and OSP in young primary school children.

## Conclusions

The presence of CHC at school entry may have negative effects on OSP at the end of first grade. A consequence-based approach, such as using the CSHCN Screener, can identify a group of children with CHC who may be particularly at risk for performing worse at school. Whether these children may benefit from early targeted medical, educational, and/or social support interventions should be addressed in the future. Long-term follow-up of future school performance is highly desirable in children with CHC to estimate the burden on this population at-risk.

## Supporting information

S1 Table Overview of the identification of diagnoses and conditions of chronic health conditions. If one of the questions was answered positively by parents, the child was assumed to have a diagnosis for this disease.
(PNG)

S2 Table Overview of how confounders were defined using the information from parental questionnaires. Confounders were used for the analysis of the effect of chronic health conditions on overall school performance.
(PNG)

S3 Table Characteristics of children within each group of chronic health condition. Characteristics of children with special health care needs (alone or in combination with a doctor’s diagnosis/condition), children with a diagnosis of a chronic disease (only), and children with no chronic health condition.
(PNG)

S4 Table Frequencies of children with different characteristics for special health care needs. Frequencies of children with special health care needs, its individual indicators, and the number of qualifying indicators of the CSHCN^1^ Screener (N = 1145, missings not shown).
(PNG)

S1 Public Use File The public use file consists of the following variables used in primary analysis: Socioeconomic status (socEcoStat), gender, overall school performance (osp), migration background (mig), one-parent family (oneParFam), school location urban/rural, (location), breastfeeding (breastfeed), active and interactive behaviour (actInterBeh), early musical education (musicEdu), television in child room (tvChildroom), outdoor activities (outdoor), smoking in house hold (smokingHH), multiples (multiples), school class (schoolClass), chronic health condition (chc). (TXT)

## Abbreviations

CHC: Chronic Health Condition
CSHCN: Children with Special Health Care Needs
OSP: Overall School Performance
PHE: Preschool Health Examination
PQ: Parental Questionnaire
ikidS: ich komme in die Schule (German for “I am starting school”)
