# Supplementary figures and images for: Chronic health conditions and school performance in first graders: A prospective cohort study
Source: PLoS One. 2018 Mar 27;13(3):e0194846. doi: 10.1371/journal.pone.0194846 (PMC5870990; doi:10.1371/journal.pone.0194846)

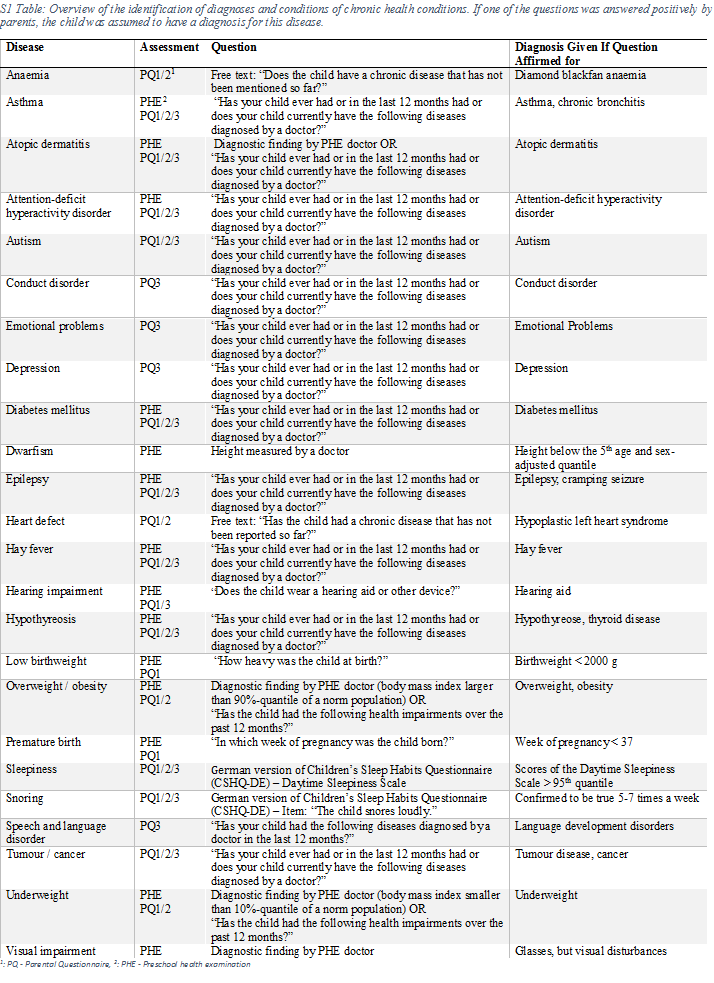

Supplement: S1 Table — If one of the questions was answered positively by parents, the child was assumed to have a diagnosis for this disease. (PNG) [file pone.0194846.s001.png]

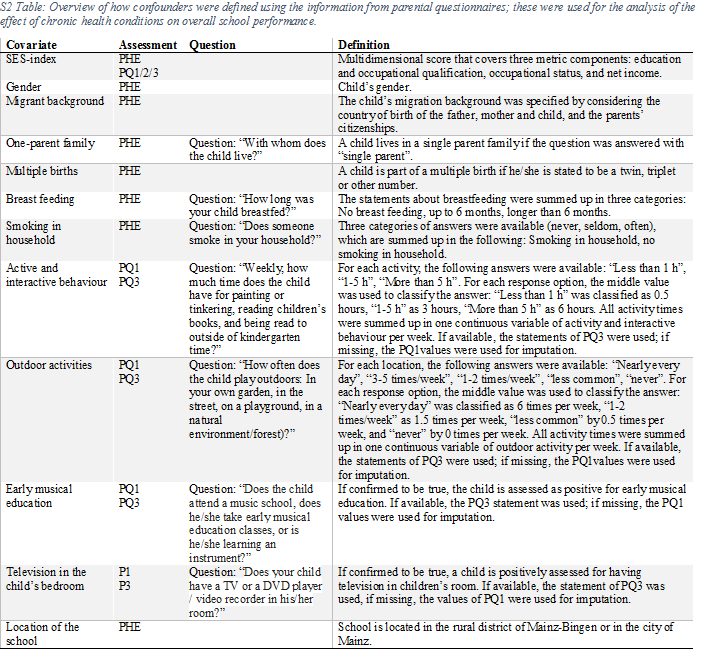

Supplement: S2 Table — Confounders were used for the analysis of the effect of chronic health conditions on overall school performance. (PNG) [file pone.0194846.s002.png]

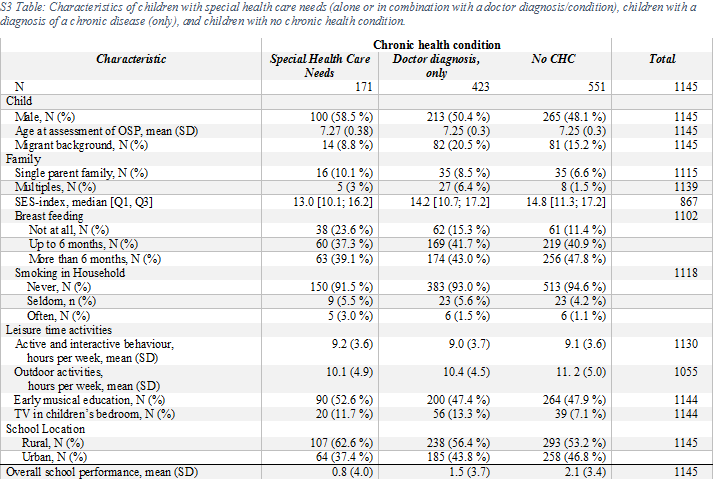

Supplement: S3 Table — Characteristics of children with special health care needs (alone or in combination with a doctor’s diagnosis/condition), children with a diagnosis of a chronic disease (only), and children with no chronic health condition. (PNG) [file pone.0194846.s003.png]

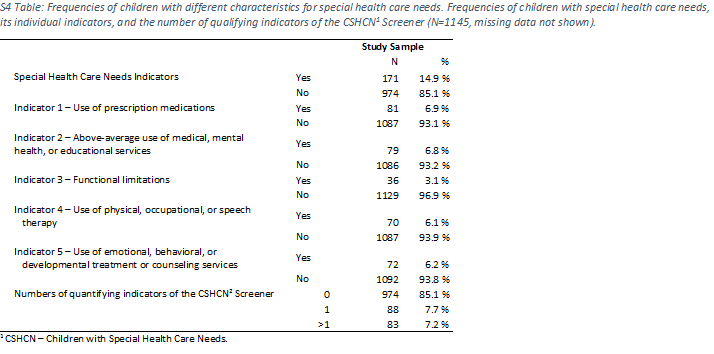

Supplement: S4 Table — Frequencies of children with special health care needs, its individual indicators, and the number of qualifying indicators of the CSHCN1 Screener (N = 1145, missings not shown). (PNG) [file pone.0194846.s004.png]
